# Kinesio Taping as a Therapeutic Tool for Masticatory Myofascial Pain Syndrome—An Insight View

**DOI:** 10.3390/ijerph20053872

**Published:** 2023-02-22

**Authors:** Ahmed Shaher Alqahtani, Sameena Parveen

**Affiliations:** Department of Maxillofacial Surgery and Diagnostic Sciences, College of Dentistry, Jazan University, Jazan 45142, Saudi Arabia

**Keywords:** masticatory myofascial pain syndrome, kinesio taping, myofascial trigger points, therapeutic tool

## Abstract

Myofascial pain syndrome (MPS) is thought to stem from masticatory muscle hypersensitivity. Masticatory myofascial pain syndrome (MMPS) is characterized by multiple trigger points (MTrPs), also known as hyperirritable points, in taut bands of affected muscles, regional muscle pain, or referred pain to nearby maxillofacial areas like teeth, masticatory muscles or the temporomandibular joint (TMJ). Muscle stiffness, reduced range of motion, muscle weakening without atrophy, and autonomic symptoms may accompany regional discomfort. Multiple treatments have been utilized to reduce trigger points and mandibular function restrictions. As a result of these incapacitating symptoms, MMPS can significantly impair many elements of quality of life. The application of Kinesio tape (KT) is a non-invasive method of treating dormant myofascial trigger points. Utilizing the body’s innate capacity for self-repair, this technique entails taping specific regions of the skin. KT alleviates discomfort, decreases swelling and inflammation, enhances or suppresses motor function in the muscles, stimulates proprioception, promotes lymphatic drainage, stimulates blood flow, and expedites tissue recovery. However, studies conducted to assess its effects have frequently yielded contradictory results. To the best of our knowledge, just a few research has looked into the therapeutic effects of KT on MMPS. The purpose of this review is to determine the efficacy of KT as a therapeutic tool for regular treatment or as an adjunct to existing therapy for MMPS based on the evidence presented in this review. To establish KT as a reliable independent treatment option, additional research is necessary to confirm the efficacy of KT techniques and applications, specifically randomized clinical trials.

## 1. Introduction

Myofascial pain syndrome is a multifactorial musculoskeletal disorder with a wide range of possible regionalized manifestations in terms of symptoms and severity. Masticatory myofascial pain syndrome (MMPS) is characterized by multiple trigger points (MTrPs), also known as hyperirritable points, in taut bands of affected muscles, regional muscle pain, or referred pain to nearby maxillofacial areas like teeth, masticatory muscles or the temporomandibular joint (TMJ). MMPS is frequently diagnosed in clinical practice, with a prevalence as high as 30% and a ratio of up to 85% [1]. Despite their limitations, the Revised Diagnostic Criteria for Temporomandibular Disorders are the most widely used diagnostic guidelines for masticatory myofascial pain syndrome [2,3,4,5].

A physical examination can quickly identify myofascial trigger points (MTrPs) inside a tight band of the afflicted muscle, probing which may elicit referral pain and a local twitch reaction. Muscle stiffness, reduced range of motion, muscle weakening without atrophy, and autonomic symptoms may accompany regional discomfort [6,7,8]. As a result of these incapacitating symptoms, MMPS can significantly impair many elements of quality of life. Although the specific cause of MMPS is unknown, various risk factors for the disease’s development have been proposed, including acute muscle overload, trauma, poor posture, and psychological stress [9,10]. As a result, a single therapy approach rarely entirely resolves the symptoms. Each patient needs individualized and multisectoral care to restore muscle strength and flexibility, deactivate MTrPs, and sustain pain management [11,12]. MMPS treatment strategies are primarily divided into invasive and non-invasive techniques. Pharmacotherapy, acupuncture, electrotherapy, cold spray, manual therapies, stretching, ischemic compression, intraoral appliance therapy, and massage are non-invasive treatment techniques. Although invasive procedures such as trigger point injections and dry needling are preferred, they are not without risk [13,14,15]. Predisposing variables must be eliminated, and structured exercise programs must be implemented for optimal MMPS management [16,17]. Even with numerous therapies, the pain syndrome may not be alleviated.

The Kinesio Taping is a therapeutic tool that is used by rehabilitation specialists in all programs, including pediatric, geriatric, orthopedic, neurological, oncology, and others, as well as levels of acute care, inpatient rehabilitation, outpatient rehabilitation, home care, and Day Rehab [18,19,20]. Among the many possible applications of KT are the following: repairing damaged or weak muscles; accelerating recovery from musculoskeletal injuries; facilitating or inhibiting muscle activity; an increasing pain-free range of motion (ROM); boosting proprioception; providing soft tissue support; alleviating headaches and sinus pain; improving joint alignment; reducing swelling and edema; minimizing pain; managing lymphedema; [21,22,23,24,25,26,27]. By microscopically lifting the skin, the KT therapeutic taping technique alleviates pain and facilitates lymphatic drainage. This lifting effect creates convolutions in the skin, expanding the interstitial space and reducing inflammation in the affected area. The Kinesio Taping (KT) technique has recently gained popularity for the treatment of sprains and strains sustained during athletic activities, postoperative complications, and various types of musculoskeletal injuries, including the management of pain. KT is a non-invasive procedure for releasing dormant MTrPs. This technique involves taping specific areas of the skin to stimulate the body’s natural ability to repair itself [28,29]. Kinesiology taping’s effectiveness in alleviating the symptoms of musculoskeletal disorders has been widely documented and lauded, making it a go-to treatment option for both professional athletes and the public. KT as a therapeutic approach was initially published in the literature in 1969 as a valuable measure for elbow joint rehabilitation [30,31,32]. KT has now acquired acceptance as a beneficial method in the treatment of acute and chronic musculoskeletal issues, such as pain, paresthesia, joint instability, and edema in many regions of the musculoskeletal system [33]. The tape’s tension gradually elevates the skin, perhaps increasing lymphatic flow and diverting it to less congested routes [34,35]. The significant effects of this technique are the restoration of muscular tension and improvements in small vessel blood circulation and lymph flow [28,36]. Kinesio Taping (KT) has gained popularity for treating MTrPs because of its non-invasiveness, painlessness, and decreased time commitment [37]. However, studies conducted to assess its effects have frequently yielded contradictory results. To the best of our knowledge, just a few research has looked into the therapeutic effects of KT on MMPS [38,39]. The purpose of this review is to present studies that determined the efficacy of KT as a complementary therapeutic tool to standard therapy for MMPS treatment to improve treatment outcomes.

## 2. Search Study and Selection Criteria

The relevant literature was comprehensively searched through PubMed, Saudi Digital Library, Science direct, and Medline from start until April 2022 using the following terms “Kinesio taping”, “Kinesiology”, “kinesiology tape”, “Ktape”, “Taping” in conjunction with “Myofascial Pain Syndrome” or “MPS” or “MPDS” or “Musculoskeletal pain” or “Musculoskeletal disorder” to construct this review. A manual search of bibliographic references in major papers and reviews, as well as Google Scholar, yielded additional studies. Abstracts and papers written in languages other than English were excluded. Additional articles were uncovered by checking the reference lists of the articles that were already examined. The specific question addressed was, “What are the possible applications of KT for masticatory myofascial pain dysfunction?”.

## 3. Kinesio Taping

### 3.1. Background of Kinesio Taping

Dr. Kenzo Kase, a Japanese chiropractor and acupuncturist, created Kinesio taping (KT) in 1973 to support the musculoskeletal system without excessive immobilization [40,41]. Initially, the objective was to minimize swelling by regulating edema, supporting soft tissues, protecting joints, and reducing inflammation-caused heat [21,42]. The main objective was to extend the benefits of manual therapy from the clinic to daily life at home and in other settings. After the Japanese athletics team used KT in the 1988 Olympics in Seoul, it attracted worldwide recognition and was imported to the US, where it gained popularity [29]. MMPS, subacromial syndrome, hemiplegic shoulder, lymphedema, tendinitis, lateral epicondylitis, patellofemoral pain syndrome, and knee osteoarthritis are treated by KT [30,34,35]. Previous research demonstrated post mandibular fracture surgery; KT reduced post-surgery swelling in the first two days [43]. Despite its novelty as a tape method, few studies have examined it. Effectiveness in treating MPS. The lack of data makes it hard to tell how different types of KT work on MMPS.

### 3.2. Characters of Kinesio Taping

Kinesio is woven cotton and elastic tape used to manage movement and accomplish functional goals. Kinesio Tape’s elastic fibers might be cotton or polyester. Tape is applied to the paper backing using an acrylic adhesive and 10–15% stretching [44,45]. In Kinesio Taping, this strain is called “paper off tension”. This tape’s heat-sensitive acrylic adhesive and cotton-wrapped elastic core lessen the risk of latex allergy in children. The non-medicinal tape is water-resistant and may remain effective for 3 to 5 days. Waved designs may alternate proprioception and somatosensory inputs. The elastic tape may be easily trimmed to fit any body alignment [29]. Kinesio Tape may stretch to 120–150% of its original length without stretching.

### 3.3. Types of Kinesio Taping

For humans, there are four Kinesio Tape varieties and two for animals. Clinicians pick tape based on the patient’s purpose and skin condition. Classic or Performance+ Tape is suggested for beginners. KT varies in size and form [21,23,42].

Types of KT and its features and tape selection based on types of applications are mentioned in Table 1.

## 4. Mechanism of Action of Kinesio Taping

**Figure 1:**
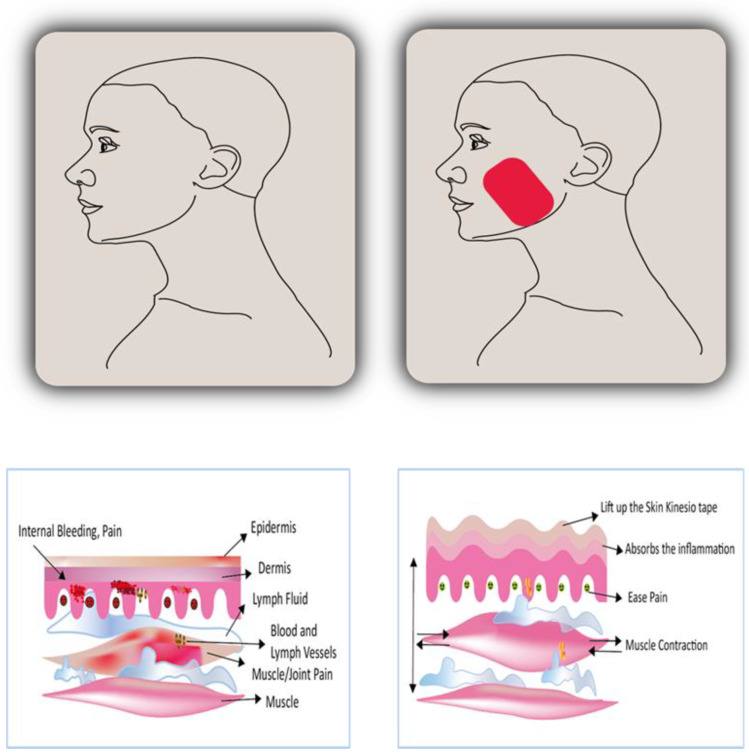
These two images demonstrated how KT is applied to soft tissue and its mechanism. The lesion site, such as a taut band or bruise, may have bleeding, pressure, and lymph fluid build-up which will then cause pain before taping. The fluid drainage is aided by the space-lifting mechanism after taping as shown. The muscles’ ability to move will then be enhanced as the pressure and inflammatory factors are decreased [29].

The skin is the body’s largest sensory organ and a key conduit for eliciting the appropriate motor responses. The required therapeutic motor impact can be achieved by applying the KT to certain skin sensory receptors [29,30]. So, it alleviates discomfort, decreases swelling and inflammation, enhances or suppresses motor function in the muscles, stimulates proprioception, and promotes lymphatic drainage. When placed on the skin with minimal strain, KT raises the region under the skin and soft tissue to increase subcutaneous space, stimulate blood and lymph fluid circulation, expedite tissue recovery (Figure 1) and channel fluid flow away from and into the damaged area to speed the recovery of wounded tissue. Varying the application and tension of the tape influence the body’s natural healing processes [29,44].

Pain and abnormal sensations are minimized, muscles are supported, subcutaneous lymphatic fluid and blood are emptied, and joint derangement is repaired. This product lifts skin, improves space between muscles and skin, and reduces pressure. Reduced pressure diminishes subcutaneous pain receptor activation, allowing pain-free movement [29,46]. Previous research showed the effect of tape on blood circulation to confirm that regular compression and decompression of the lymphatic system boosted flow and circulation [29]. Negative-pressure pumping directs fluid from the superficial to deeper levels of the one-way lymphatic system beneath the skin [29,43]. Muscle contraction and relaxation help create negative pressure. Intercellular junction shutters close as edema increases the interstitial pressure in the lymphatic system. Muscular contraction and relaxation, massage, and compression clothing may vary the pressure in each area. Lymph and interstitial fluid recirculation reduce swelling and pain. Lymph edema drains lesions and promotes tissue repair, improving circulation [47,48].

**Figure 2:**
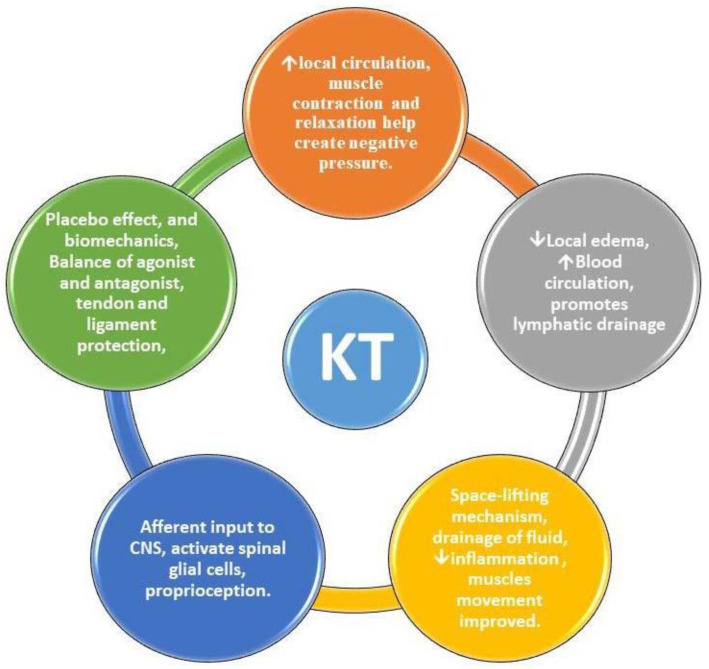
Effects of Kinesio taping on masticatory myofascial pain syndrome. CNS—central nervous system; KT—Kinesio taping.

Taping may affect proprioception, placebo effect, and biomechanics. Patients with patellofemoral pain syndrome may benefit from further KT [49]. Gate control theory may explain KT’s pain-management benefits. Touch A-fiber diameter and conduction velocity are higher than pain A- and C-fibers. Mildly touching afferent skin receptors may activate glial spinal cells. The spinal cord blocks pain conduction to the brain [11]. Although KT does not replace the need for exercise, it is recommended as an adjunctive strategy for the short term in the treatment of pain [45]. Therefore, the KT tape is not to be used as the sole form of treatment, but rather as an alternative or complementary treatment when an immediate and short-duration effect by application is desired. Pairing with other methods and tools is recommended including manual therapy, muscle strengthening, electrothermal, and phototherapy [50]. Kinesio taping may be the best treatment option for patients with myofascial pain syndrome, but more high-quality RCTs are needed to determine this at post-intervention and follow-up stages. Schematic diagrams represent the effects of KT application for MMPS in Figure 2.

## 5. Clinical Application of Kinesio Taping for Myofascial Pain Syndrome

KT, when used in conjunction with other therapy modalities, may provide additional advantages such as enhanced proprioceptive feedback and enhanced joint stabilization. To be able to recommend KT to a patient, a practitioner has to be aware of the patient’s previous medical history, the nature of the patient’s occupation or activity, as well as the biomechanics of the injury, and the duration of the damage. A complete physical examination will include inspection, palpation, and active and passive ranges of motion (ROM), as well as the identification of the MTrPs. There have been a number of investigations on the effectiveness of KT for the management of myofascial pain syndrome in TMJ patients. According to a systematic review and meta-analysis, the kinesiology tape had a significant effect on the masseter muscles, with tremendous pain relief assessed with VAS after using the tape for one week in comparison to other interventions occlusal splint, compressions to deactivate MTrPs, and counseling are some of the treatment options in people who suffered from temporomandibular disorders (TMDs). The inverse variance approach was used throughout the process of doing the meta-analysis. The mean value that was displayed on the visual analog scale was regarded to be the intervention effect measure. measure. The experimental group had 1.4 points of pain intensity decrease in VAS, with a *p*-value = 0.013. The conclusions of the research were, however, restricted due to the low number of studies and the inherent biases in those investigations [51].

The efficacy of KT for treating temporomandibular joint (TMJ) problems were studied in a single-blind, randomized, controlled experiment undertaken by Coskun Benlidayi and colleagues [52]. Patients with TMDs were divided into two groups, one for the trial and the other as a control. Fourteen participants in the study got KT in addition to counseling and jaw exercises, whereas another fourteen participants in the control group received just therapy and exercise alone. Before beginning therapy, the first and sixth weeks measured the efficiency of the patient’s chewing, as well as the patient’s self-reported functional limitations. The biobehavioral questionnaire was completed twice, once at the beginning and again at the end of the study. Active mouth opening increased more in the treatment group compared to the control group. A substantial improvement in the visual analog scale (VAS) for TMJ, masticatory efficiency of mastication, and functional restriction was seen in the experimental group but not in the control group. The study showed that the experimental group’s subjective treatment effectiveness was greater than that of the controls. The experimental group had substantial decreases in pain (*p* = 0.001), sadness (*p* = 0.006), and disability ratings (*p* = 0.01), but not the control group. To sum it up, when it comes to TMDs, KT is more beneficial than counseling and exercise by itself (or even in combination) [24].

Patients with latent MTrPs in the sternocleidomastoid muscle (SCM) were taped using Kinesio taping to see how that affected their myofascial discomfort and temporomandibular joint range of motion. A total of 42 individuals between the ages of 20 and 30 took part in the study (male 17, female 25). One group received KT, and the other received a placebo. The SCM was Kinesio-taped three times a week for two weeks. The amount of discomfort felt while pressing on a tight band or nodule was recorded and analyzed. VAS and pressure pain threshold (PPT) was used to assess the degree of discomfort. The temporomandibular joint’s range of motion was assessed. Each participant’s temporomandibular joint range of motion, VAS, and degree of discomfort were evaluated pre- and post-treatment. It was discovered that pain in the SCM muscle was relieved after Kinesio taping was applied to subjects with latent MTrps and restriction of the ROM of the TMJ. This was demonstrated by the fact that the VAS score and PPT significantly (*p* < 0.01 and *p* < 0.05, respectively) decreased. The TMJ’s ROM has significantly increased (*p* < 0.05). These findings showed that Kinesio taping reduced pain in latent MTrPs that was brought on by overexerting the SCM muscle, and that the ROM of the TMJ also changed as overexerting the SCM muscle decreased. The control group, on the other hand, exhibited no change between the times of the taping and before. Significant differences in VAS and PPT scores as well as TMJ ROM were found when comparing the groups (*p* < 0.05). Hence it was concluded KT could be used to treat latent MTrPs [34].

Another illustration of this is a comparative study carried out showed that, after treatment, there was not a substantial difference discovered between the two categories with respect to values on the VAS or measurements of mouth opening; however, the values achieved for the pain threshold for the temporal muscle were higher in the Kinesio group. It was an easy-to-apply non-invasive procedure that did not stop regular oral activities, in addition to minimizing the amount of discomfort experienced [53]. Ristow and associates examined the efficacy of KT on postoperative edema, discomfort, and trismus in 26 patients with open reduction and internal fixation of mandibular fractures. Within the first two days after surgery, the administration of KT using the lymphatic approach had the ability, according to this research, to cut the occurrence of edema and reduce turgidity by at least 60%. However, there was no discernible impact on the amount of pain relief experienced [43]. Another research that aimed to elucidate the impact of KT on third-molar surgery-related postoperative morbidity, found that KT decreased edema, trismus, and discomfort, which ultimately resulted in a lower morbidity rate after surgery [54]. The researchers drew attention not only to the positive outcomes that may be achieved with KT but also to the fact that it is risk-free and requires just a little expenditure.

Kijak and their team examined patients suffering from temporomandibular problems to see if the KT approach and the deactivation of trigger points might successfully eliminate pain without using pharmaceuticals. The research was carried out on a total of sixty patients with an age range of 18 to 35 years. The participants were randomly split into groups of two, each consisting of thirty individuals. KT has been applied actively to the members of Group KT (there were 15 males and 15 women in this group). Physiotherapy was performed on Group MTrP, which included the ischemic compression approach for the release of trigger points. There was a total of 16 females and 14 males in this group. The results show that MTrP inactivation and the KT technique had significant therapeutic analgesic effects throughout pain-related functional abnormalities of the muscles involved in mastication. After utilizing the KT strategy, which increased the analgesic effect in patients with dysfunction, the researchers noticed that the treatment positively impacted the patients. There was no discernible effect of either the patient’s gender or age on the therapy outcomes. In addition, they concluded that there is a need for the development of algorithms for diagnosing and treating oral facial pain, as well as how dentists and physiotherapists contribute to the process [28].

Previous studies prospectively compared the early results of Kinesio tape (KT) to those of corticosteroid injection (CSI) and the rest-and-medication group (RMG) for the treatment of lateral epicondylitis. Nirschl scores were used to categorize patients, and the VAS, QDASH, and the Turkish adaptation of the Patient-Related Elbow Evaluation were used for further evaluation (PREE-T). All groups showed statistically significant gains by the end of the second week. The functional scores for those in the RMG and CSI groups decreased slightly from week to week, and only KT remained effective throughout the entire study. At week four, the average QDASH score for the KT group was 18.1 (4.5–35), the VAS score was 2 (1–3), the VAS score for resisted wrist extension was 4 (2–4), and the Nirschl score was 2 (1–3). It was found that the KT group performed better than the RMG in all four of these measures, *p* = 0.035, *p* = 0.035, *p* = 0.029, and *p* = 0.035, respectively [39].

The effectiveness of trapezius-muscle kinesio taping (KT) in addition to TPI (TPI of 1 mL of 20 mg betamethasone dipropionate combined with 3 mL of 2% lidocaine and 1 mL of saline solution) for the treatment of MPS was investigated in a separate study. Fifty patients diagnosed with MPS were divided into two groups of 25 individuals using a random number generator. Group 1 TPI plus KT; TPI plus sham KT in Group 2. At pretreatment, one, and three months posttreatment, patients filled out a VAS and a Neck Disability Index (NDI). VAS and NDI scores at 3 months were significantly lower in Group 1 versus Group 2 (*p* < 0.05). Treatment with TPI and KT on the trapezius muscle for MPS has shown promise in halting the chronic process, preventing recurrence, and breaking the pain cycle. Due to its limited role in the long-term management of MPS, MTrP injection runs the risk of creating dependence in patients when used as the primary therapy. The combined therapy appears to generate a more influential outcome in the long run, than monotherapy, in alleviating pain and reforming functional amelioration in the management of MPS [55].

The results of KT and dry needling (DN) for the treatment of trapezius muscle myofascial pain syndrome were compared in another recent study. A blinded evaluator took readings on a Visual Analog Scale (VAS), Pressure Pain Threshold (PPT), Neck Disability Index (NDI), and Global Perceived Effect Scale (GPES) at the beginning and end of the second week. The pain, disability, and global effects of MPS treatment are significantly better for those who undergo KT and DN as opposed to those who do not (*p* < 0.001 for all). Supplementing an exercise regimen with DN or KT applications improved outcomes for patients with MPS. Comparative effectiveness between DN and KT was not observed [56].

Among adult patients with cervical dystonia (CD), the application of KT after botulinum toxin (BoNT) injection did not affect the severity of dystonia but did improve quality of life (*p* < 0.05, *p* = 0.03). The Toronto Western Spasmodic Torticollis Rating Scale (TWSTRS), comprising Torticollis severity, Disability, and Pain scales, was used to quantify the severity of CD. The Craniocervical Dystonia Questionnaire was used to assess the quality of life (CDQ4) [57]. However, we need more studies looking into the impact of KT before and after BoNT injection in MMPS.

Despite extensive research, KT has only recently been used in the maxillofacial region. Additionally, little is known about the potential impact on MMPS. KT has not been proven to be an effective stand-alone treatment for MMPS, but the studies we’ve highlighted in this review suggest it may be useful as an adjunct to conventional care. To be accepted as a viable independent treatment option, KT must be thoroughly explored in future studies, especially randomized clinical trials.

## 6. Limitations and Research Gap

KT has only recently been used in the maxillofacial region, and there haven’t been many studies published on this topic; it is still relatively new in the field of dentistry. Furthermore, not much is known about how KT can affect the TMJ and masticatory muscles. In addition, the current research has numerous flaws because, despite the fact that the sample size was sufficient for a statistical analysis that successfully identified significance in comparisons, it’s more reasonable to back the findings of this research with trials with a larger sample size. Some researchers did not include a placebo group. The usefulness of KT, if any, should be clarified via placebo-controlled research with more significant sample numbers independent of the placebo effect. So, more studies are required in this field that should strive to use KT over other methods of intervention in the maxillofacial region. In addition, there is a specific limitation in using KT under the circumstances like open wounds, deep vein thrombosis, allergy, fragile skin, and active malignant sites. Self-application of tape, however, can be challenging due to a lack of familiarity with the taping procedure, the need to use both hands, the placement of the MTrP, and a lack of knowledge of anatomy or biomechanics. As a result, the vast majority of people can’t tape independently.

## 7. Conclusions

Treatment of MMPS may be challenging due to its complex pathology and underlying peripheral and central neural mechanisms, particularly during the chronic phase. The inability to recognize trigger points, a lack of experience with the taping technique, and a lack of knowledge of anatomy or biomechanics are some other obstacles to self-taping. From a variety of treatment options (such as manual therapies, massage, electrophysical agents, medical care, electrotherapy, cold spray/stretching, ischaemic compression, and acupuncture), the choice of kinesiology taping remains up to the individual practitioner and clinic policy. Healthcare professionals may, however, continue to view kinesiology taping as a practical therapeutic non-pharmacological approach for MMPS management in light of the current evidence. It allows for a significant reduction in edema and discomfort without endangering the patient’s metabolism or digestive system, is simple to perform, affordable, and less traumatic. Finally, studies included in this review demonstrated that, despite its limitations, KT can be considered an adjunctive therapeutic tool to standard therapy for the treatment of MMPS and has not been shown to be a valid independent treatment option. It’s still unclear whether Kinesio taping is more effective in the long run than other treatments. Future studies are necessary to confirm the effectiveness of KT techniques and applications, particularly randomized clinical trials, to establish KT as a reliable independent treatment option.

## Figures and Tables

**Table 1:** Types and Features of Kinesio taping.

S. No.	Types of Kinesio Taping	Features	Reference
1.	KinesioTex Classic	Universal and best option since it can be used in any situation and is most suited for healthy skin.	[23]
2.	KinesioTex Performance+	Polyester and cotton blends are excellent for delicate skin when more significant tape tensions are needed.	[21]
3.	KinesioTex Gold	For low-tension applications and uses a specific distribution of glue accessible exclusively to qualified specialists that provide high adhesion without needing an enormous surface area.	[23]
4.	KinesioTex Gold Light Touch Plus	used for short-term treatments and is often used on youngsters and the elderly with sensitive skin.	[21]
5.	Kinesio Equine	It can be applied straight to horsehair and has a flavor that animals would not eat.	[33]
6.	Kinesio Canine	For dogs, it works nicely with their hair.	[33]

## Data Availability

Not applicable.
